# Changes in glutamic oxaloacetic transaminase 2 during rat physiological and pathological cardiomyocyte hypertrophy

**DOI:** 10.1186/s12872-023-03648-3

**Published:** 2023-12-05

**Authors:** Xin Liu, Xiaolu Li, Haotan Zhou

**Affiliations:** 1 Department of Pediatric Cardiac Center, Beijing Anzhen Hospital, Capital Medical University, Beijing, 100029 China; 2 Experimental Research Center, Beijing Institute of Heart, Lung and Blood Vessel Diseases, Beijing Anzhen Hospital, Capital Medical University, Beijing, 100029 China; 3 Department of Pathology, Beijing Anzhen Hospital, Capital Medical University, Beijing, 100029 China

**Keywords:** Physiological cardiomyocyte hypertrophy, Pathological cardiomyocyte hypertrophy, Glutamic oxaloacetic transaminase, Congenital heart disease

## Abstract

**Background:**

Physiological and pathological cardiomyocyte hypertrophy are important pathophysiological processes of adult congenital heart disease-associated ventricular hypertrophy. Glutamic oxaloacetic transaminase (GOT) is a vital marker of myocardial injury. This study aimed to investigate the changes in GOT levels during physiological and pathological cardiomyocyte hypertrophy in rats.

**Methods:**

RNA-seq analysis and colorimetric methods were used to evaluate the changes in GOT mRNA and activity, respectively. GOT2 protein expression was detected by western blotting and immunofluorescence. Hematoxylin-eosin and wheat germ agglutinin methods were used to observe changes in rat cardiomyocyte morphology.

**Results:**

In juvenile rat hearts, GOT mRNA expression and activity, and GOT2 protein level increased with age-related physiological cardiomyocyte hypertrophy; however, GOT2 protein level was reduced in hypoxia-induced pathological cardiomyocyte hypertrophy.

**Conclusions:**

GOT2 may regulate physiological and pathological myocardial hypertrophy in rats. We speculated that the low GOT2 level contributed to the rapid occurrence of pathological cardiomyocyte hypertrophy, causing strong plasticity of right ventricular cardiomyocytes in the early postnatal period and heart failure in adulthood.

**Supplementary Information:**

The online version contains supplementary material available at 10.1186/s12872-023-03648-3.

## Background

Cardiomyocyte hypertrophy, including physiological and pathological cardiomyocyte hypertrophy, is a crucial pathophysiological process of adult congenital heart disease (CHD)-associated ventricular hypertrophy [1–4]. The most common adult CHD is atrial septal defect (ASD). The typical pathophysiological changes in ASD include right ventricular hypertrophy with less significant alterations in the left ventricle. Previous studies have indicated greater plasticity of the right ventricular myocardium in the early postnatal period. In previous studies, cardiomyocyte volume in the right ventricle increased remarkably in children with Tetralogy of Fallot (TOF) [5–7]. A mouse model of pulmonary artery banding, used to simulate changes in the right ventricle of TOF, revealed that cardiomyocyte hypertrophy of younger mice is more evident than older ones further [8]. Recent studies on the regulation of cardiomyocyte hypertrophy unlocked that methyltransferase (METTL) and Akt showed opposing changes during physiological and pathological cardiomyocyte hypertrophy [9–12]. However, the molecules involved in both physiological and pathological cardiomyocyte hypertrophy are complex and diverse, and their roles have not been still fully elucidated.

Glutamic oxaloacetic transaminase (GOT or AST), closely related to myocardial function, is an essential myocardial injury biomarker [13–15]. GOT isozymes, GOT1 and GOT2, are located in the cytoplasm and mitochondrial matrix respectively, and participate in nicotinamide-adenine dinucleotide phosphate (NADPH) oxidative phosphorylation to produce ATP through the malate-aspartate shuttle in cardiac energy metabolism [16, 17]. A clinical study indicated that the activity ratio of serum GOT/alanine transaminase was negatively correlated with the incidence of cardiovascular disease in a healthy Japanese population [18]. The excessive increase in serum GOT activity was associated with a decline in the survival rate of children with cyanotic CHD and right ventricular hypertrophy, such as TOF [14]. In adult animal experiments, angiotensin II (Ang II)-induced pathological cardiomyocyte hypertrophy was associated with decreased GOT2 protein expression, but not with GOT1. Notably, the experimental cardiac hypertrophy may act as a specific stimulus for GOT during myocardial malate-aspartate shuttle [19, 20]. Moreover, hypoxia is not only a typical symptom of cyanotic CHD but a critical cause of disease progression. The results of a perinatal mouse model confirmed that chronic hypoxia inhibited heart development, which presented as an increase in the heart-weight ratio [21, 22]. These studies suggested that GOT2 was correlated with right ventricular hypertrophy; however, GOT2 alterations in age-related physiological and hypoxia-induced pathological cardiomyocyte hypertrophy during childhood remain unexplored.

This study aimed to determine the changes in GOT2 during physiological or hypoxia-induced pathological cardiomyocyte hypertrophy. To this end, we assessed age-related changes in GOT2 expression of juvenile rat hearts, the correlation between GOT activity and physiological cardiomyocyte hypertrophy in rat right ventricle, and GOT2 expression changes in hypoxic cardiomyocyte hypertrophy to provide a novel idea and research target for promoting physiological cardiomyocyte hypertrophy, suppressing pathological cardiomyocyte hypertrophy, and accelerating the heart’s transition to left ventricular dominance after birth.

## Methods

### Day-age choice and tissue processing in immature rats

Previous studies have reported a transition in the rat heart from cardiomyocyte proliferation to cardiomyocyte hypertrophy between the 3rd and 4th days following birth. Subsequently, cytokinesis decreases, and cardiomyocytes primarily grow in a hypertrophic manner. However, the myocardial nuclear division of Sprague Dawley (SD) rats decreased significantly on the 10th day [23]. Compared with previous studies, the positive rates of ki-67, a nuclear division biomarker, were similar in humans within 0–3 months and in SD rats within 28 days (0.55% vs. 0.5%) after birth; thereafter, the ki-67 almost disappeared in humans and rats [8]. In addition, γ- linolenic acid, a type of fatty acid in breast milk, is crucial for the metabolic maturation in the heart development of suckling mice [24]. Therefore, as indicated in Supplementary Fig. 1, we selected 5-, 10-, and 20-day-old suckling mice to represent the three turning points.

Juvenile male SD rats in the 5-, 10-, and 20-day-old groups (*n* = 3–5 each) were purchased from Beijing Vital River Laboratory Animal Technology Co., Ltd. The juvenile rats were sacrificed under pentobarbital sodium (5.4 g/kg) treatment [25, 26]. We quickly dissected and opened the abdominal and chest cavities; collected the heart, lung, and liver; and washed them with phosphate-buffered saline (PBS) at 4 °C. After absorbing excess PBS with filter paper, the freshly collected tissues were stored at − 80 °C or fixed in 4% paraformaldehyde solution for 24 h to prepare paraffin sections.

### Gene database search

Using the National Center for Biotechnology Information (NCBI) gene database, we obtained the RNA-seq data for GOT1 and GOT2 in 11 organs of female and male Fischer 344 rats aged 2, 6, 21, and 104 weeks old, wherein each week-old group (*n* = 8) corresponds to infancy, adolescence, adulthood, and old age, respectively [27]. We selected RNA-seq data from the heart, liver, brain, kidney, adrenal gland, and lung for statistical analysis.

### GOT2 protein expression levels evaluated by western blotting

Lysis buffer was used to obtain proteins from the tissues and H9C2 cell. Denatured proteins were isolated by an 8% precast gel (Beyotime, China) and transferred onto nitrocellulose membranes. Nitrocellulose membranes were incubated with the specific primary antibodies (anti-GOT2 antibody produced in rabbit, 1:3000, SAB5701225, Sigma-Aldrich; anti-GAPDH antibody produced in mouse, 1:4000, TA-08, ZSGB-BIO) overnight at 4 °C and with secondary antibodies (1:2000) conjugated with horseradish peroxidase for 1 h at 25 °C. Finally, as previously described [28], protein bands were scanned using the FluorChem M MultiFluor System (ProteinSimple, USA).

### GOT activity determined using the colorimetric method

The rat tissues stored at − 80 °C were accurately weighed, and physiological saline and magnetic beads were added for homogenization. Subsequently, the homogenized solution was frozen three times at − 80 °C and centrifuged at 4 °C, and the supernatant was isolated for conducting tests. Following the instructions of the aspartate aminotransferase assay kit (Nanjing Jiancheng Bioengineering Institute, China) [29], we divided the samples into test and control wells, and simultaneously prepared standard concentration wells. After 30 min of reaction with the substrate solution, 20 min with the 2,4-dinitrophenylhydrazine solution, and 15 min with NaOH, the absorbance values were measured using a microplate reader at a wavelength of 510 nm.

### Hematoxylin-eosin (HE) staining

The rat heart tissue slices were sequentially placed in xylene and ethanol for dewaxing in water, and the hydrated slices were immersed in HE staining solution for 5 min. Subsequently, the stained slices were dehydrated with ethanol and made transparent with xylene. Finally, neutral resin was used to seal the slices. The slides were observed under a light microscope; the nuclei were blue, and the cytoplasm was red [30, 31]. The size of cardiomyocytes indirectly evaluated by counting the number of nuclei in images with the same magnification and area.

### H9C2 culture

The rat embryonic myocardial cell line H9C2 and DMEM cell culture medium were purchased from Shanghai Fuheng Biotechnology Co., Ltd. The complete medium contains 93% basic medium, 5% fetal bovine serum, 1% L-glutamine, and 1% penicillin/streptomycin. H9C2 cells were allowed to adhere for 24 h in the medium, following which the cells were placed in a closed chamber (2.5 L) with a bag of Anaeropack-anaero2.5 (AnaeroPack-Anaero, Japan) for 24 h (oxygen concentration: 1%) to simulate hypoxia [32]. The hypoxic model experiment included the control and hypoxic groups.

### Wheat germ agglutinin (WGA) for visualizing H9C2 morphology

After a 24-h exposure to hypoxia, the culture medium was removed by washing the cells with PBS. Next, the cells were fixed with paraformaldehyde, a drop of WGA (Sigma-Aldrich, USA) dye solution was added, and the cells were incubated in the dark for 20 min. Finally, an anti-fluorescence quenching agent containing DAPI (Servicebio, China) was used for sealing [33]. Fluorescence microscopy showed that the nuclei were blue and the WGA was green.

### In situ detection of GOT2 protein expression in H9C2 cells using immunofluorescence assay

As previously described [28], after rinsing with PBS, H9C2 cells were fixed with 4% paraformaldehyde solution for 20 min. Then, a 5% bovine serum albumin (BSA) blocking solution (0.5 g BSA, 30 μL TritonX-100 and 10 mL 0.01 M PBS) was used to block and perforate cells. After removing the excess blocking solution, H9C2 cells were incubated with the primary antibody GOT2 (1:200, Sigma-Aldrich, USA) at 4 °C overnight. The next day, the H9C2 cells were incubated with a rabbit-derived red fluorescent secondary antibody (1:500, Thermo Fisher Scientific, USA) in the dark for 90 min. All sections were restained with DAPI. Finally, immunofluorescence microscopy was used for observation and comparison. We used ImageJ to quantitatively evaluate the red immunofluorescence intensity of GOT2 in images with similar cell densities.

### Data analysis

GraphPad Prism software (version 8.0.2, USA) was used for statistical processing. Data were expressed as mean ± standard deviation (mean ± SD). An independent sample *t*-test was used to compare means between two groups. The means among multiple groups were compared by one-way analysis of variance (ANOVA) and the *Sidak* method when the data were normally distributed or the nonparametric *Kruskal-Wallis* test when the data were not normally distributed. *p* values < 0.05 were considered statistically significant [28].

## Results

### GOT expression in juvenile rat hearts was rapidly increased and GOT2 mRNA level was higher than that of GOT1

**Fig. 1:**
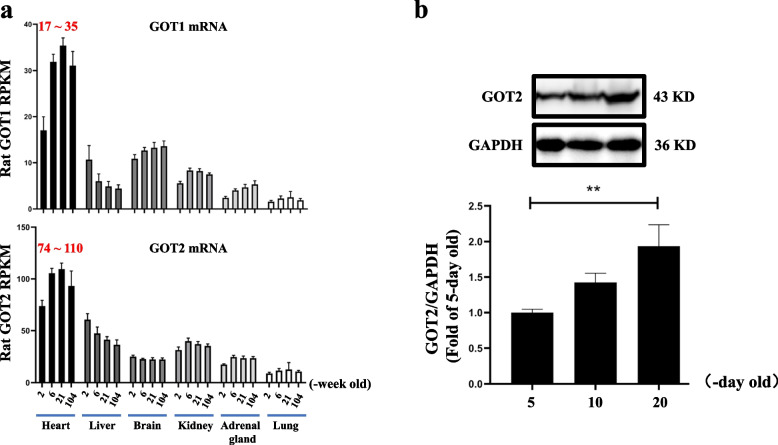
Changes in rat GOT expression in multiple tissues. **a** Age-related changes in GOT mRNA expression in different rat tissues by RNA-seq (*n* = 8). **b** GOT2 protein expression detected by western blotting in rat heart tissue (*n* = 5), full-length blots were presented in Supplementary Fig. 2. GOT: glutamic oxaloacetic transaminase. Data were expressed as mean ± SD, *p < 0.05

Considering the close correlation between GOT and myocardial function, we obtained RNA-seq data from different rat tissues at different ages (in weeks) by searching the gene database [27] and found that GOT1 and GOT2 mRNA levels in juvenile (< 6-week-old) rat hearts rapidly increased. Moreover, the GOT2 mRNA (74–110 RPKM) level was significantly higher than that of GOT1 (17–35 RPKM) in the rat myocardium (Fig. 1a). The GOT2 protein expression results detected through western blotting were consistent with the RNA-seq findings in juvenile rat hearts. Compared with the 5-day-old group, the protein level of cardiac GOT2 was dramatically enhanced in the 20-day-old group (Fig. 1b). In addition, GOT1 and GOT2 mRNAs were widely distributed in multiple organs, including rat hearts, livers, brains, kidneys, adrenal glands, and lungs, but GOT1 and GOT2 mRNAs in the different tissues other than the rat heart exhibited no significant changes and maintained low levels among the juvenile, adult (21-week-old) and aged (104-week-old) groups (Fig. 1a). These results suggested that the expression and function of GOT (particularly GOT2) were tightly linked to heart development in juvenile rats.

### GOT activity increased with the right ventricle cardiomyocyte volume in juvenile rat hearts

**Fig. 2:**
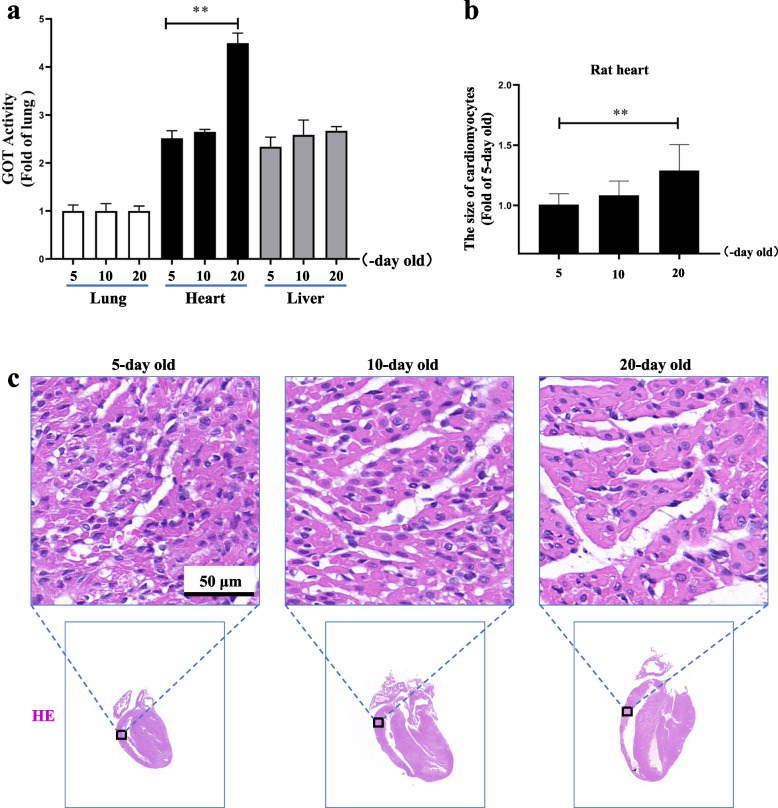
Changes in GOT activity and cardiomyocyte volume in juvenile rat hearts. **a** The colorimetric method was performed to detect the GOT activities in rat heart, liver and lung tissues (*n* = 3–5). **b, c** HE staining was used to observe the age-related physiological cardiomyocyte hypertrophy in the juvenile rat right ventricle. (*n* = 3–5). GOT: glutamic oxaloacetic transaminase; HE: hematoxylin-eosin staining. Data were expressed as mean ± SD, **p < 0.01, scale bar: 50 μm

GOT primarily participates in NADPH oxidative phosphorylation to supply energy via transaminase activity. To study the relationship between GOT function and physiological cardiomyocyte hypertrophy in juvenile rats, we further used a colorimetric method to determine the GOT activity in rat heart, liver, and lung tissues and HE staining to observe the normal age-related physiological cardiomyocyte hypertrophy in the rat right ventricle after birth. The results showed that the GOT activities in rat lungs and livers at the ages of 5, 10, and 20 days differed insignificantly; however, compared with the 5-day-old group, the cardiac GOT activity in the 20-day-old group rats was significantly increased (Fig. 2a), while the size of cardiomyocytes and heart volume were significantly increased, and hearts gradually shifted towards the left ventricular dominance (Fig. 2b and c). The above-mentioned results indicated an association between increased GOT activity and juvenile rat heart right ventricular cardiomyocyte volume, highlighting the role of GOT transaminase activity in rat age-related physiological cardiomyocyte hypertrophy.

### Reduced GOT2 protein expression in hypoxia-induced hypertrophic cardiomyocytes

**Fig. 3:**
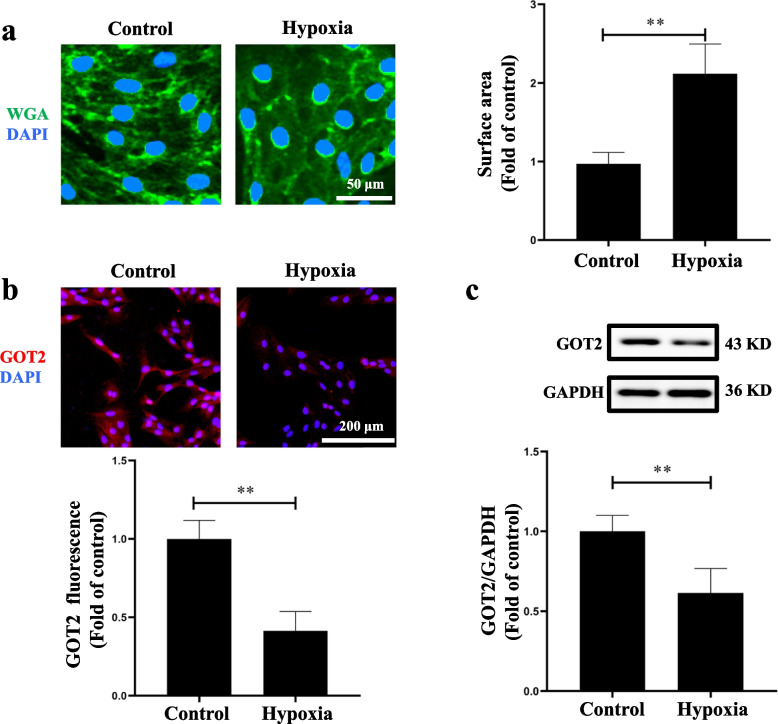
Cell surface area and GOT2 protein expression in myocardial cell line H9C2. **a** WGA staining was used to outline the edges of H9C2 cells (*N* = 3, *n* = 3), scale bar: 50 μm. **b** In situ immunofluorescence detection of GOT2 protein expression in H9C2 cells; red fluorescence represents GOT2 protein, scale bar: 200 μm. **c** Western blotting was employed to detect the GOT2 protein expression in H9C2 cells (*N* = 3, *n* = 3), full-length blots were presented in Supplementary Fig. 3. WGA: wheat germ agglutinin, a specific cardiomyocyte membrane dye; GOT2: glutamic oxaloacetic transaminase 2. N: the number of independent experiments, n: the number of duplicates. Data were expressed as mean ± SD, **p < 0.01

Hypoxia is vital in the pathological hypertrophy of the right ventricular myocardium in patients with cyanotic CHD [21, 34]. By building a hypoxic cell model, using WGA staining, immunofluorescence, and western blotting, we found that compared with the control group, the surface area of H9C2 cells exposed to hypoxia increased by 2-fold (Fig. 3a), while the red fluorescence intensity representing the GOT2 protein decreased significantly in accordance with the results of western blotting (Fig. 3b and c). These results suggested that cardiomyocyte-derived GOT2 protein expression was inhibited during hypoxic pathological cardiomyocyte hypertrophy. In other words, the reduction in GOT2 protein may be a key factor contributing to the hypoxia-induced pathological cardiomyocyte hypertrophy.

## Discussion

In this study, we initially found a positive correlation between myocardial GOT2 and age in juvenile rats through the lens of GOT, a hallmark of myocardial injury. Under physiological conditions, GOT2 may inhibit excessive cardiomyocyte hypertrophy in juvenile rats by continuously enhancing its mRNA and protein expression. Hypoxia-induced GOT2 reduction in CHD may contribute to pathological cardiomyocyte hypertrophy in children and adults with CHD.

In previous studies, the right ventricle myocardium of children with cyanotic CHD in the early postnatal period demonstrated strong plasticity [5–8]. Although some studies have shown that METTL can simultaneously regulate physiological and pathological hypertrophy, leading to significant cardiomyocyte hypertrophy [9–12], the related regulatory molecules about physiological and pathological cardiomyocyte hypertrophy of the right ventricle in the early postnatal period require further exploration. Recent studies have revealed that GOT2 was related to Ang II-induced pathological cardiomyocyte hypertrophy in adult mice. To clarify GOT2 changes in age-related physiological cardiomyocyte hypertrophy, we first observed changes in GOT during rat normal heart development. RNA-seq results showed that unlike the stable GOT mRNA expression in other organs, GOT mRNA in juvenile rat hearts increased rapidly but was relatively stable in adulthood. These results suggested that GOT played a basic and common role in rat heart, liver, brain, kidney, adrenal gland and lung cell activities and was tightly linked to heart development in juvenile rats. Moreover, GOT2 mRNA expression was higher than that of GOT1 in rat hearts. In addition, Rat heart GOT mRNA was highly expressed at all ages, which was consistent with clinical research results. GOT derived from acute injured myocardium in children after CHD surgery can cause a sharp increase in serum GOT activity [14]. Western blotting experiments further confirmed that the expression pattern of myocardial GOT2 protein was similar to that of GOT2 mRNA in juvenile rats.

To further explore the relationship between GOT function and physiological cardiomyocyte hypertrophy, we compared the changes in GOT transaminase activity and age-related physiological cardiomyocyte hypertrophy in rats and found that GOT activity increased with physiological cardiomyocyte hypertrophy. These results suggested that GOT2 may regulate physiological cardiomyocyte hypertrophy or the rapid GOT2 change in juvenile rat hearts may be related to the plasticity of cardiomyocytes.

Finally, we explored the relationship between GOT2 expression and hypoxic pathological cardiomyocyte hypertrophy. The results of cell experiments showed that hypoxia induced a decrease in GOT2 protein expression of rat myocardial cell line H9C2 and an increase in the myocardial cell surface, which was familiar with Ang II-induced GOT2 reduction in adult mice with cardiac hypertrophy [19]. Similarly, Romanowicz et al. found that chronic hypoxia inhibited juvenile mouse heart development, characterized by increased cardiac mass [21]. The above-mentioned results indicated that the inhibition of GOT2 expression may be a mechanism underlying hypoxia-induced pathological cardiomyocyte hypertrophy in cyanotic CHD. Some researchers found reactive oxygen species (ROS), could potentially modulate cardiac maturation. Considering that GOT2 is distributed in mitochondria, which are the chief consumers of oxygen and the potential source of ROS induced by hypoxia, we hypothesized that the increased ROS induced by hypoxia may be the underlying mechanism for the reduction in GOT2 expression [35–37].

## Conclusions

Based on the results of the present study, we preliminary explored GOT2 changes in age-related physiological and hypoxia-induced pathological cardiomyocyte hypertrophy in rats, providing new insights and research targets for promoting physiological and inhibiting pathological cardiomyocyte hypertrophy. Considering that physiological and pathological hypertrophy is controlled by distinct cellular signaling pathways [3], further animal experiments should be performed to delineate the specific criteria used to determine physiological and pathological hypertrophy. Further, GOT2 expression intervention experiments should be conducted to confirm the protective effect of GOT2 on cardiomyocyte hypertrophy and to provide a novel theoretical basis for the treatment of cyanotic CHD in children and adults.

### Supplementary Information

**Additional file 1.**

## Data Availability

All data required to evaluate the conclusions are presented in the manuscript and/or Supplementary Materials. The datasets analyzed in the current study are available from the NCBI repository, (https://www.ncbi.nlm.nih.gov/gene/?term=GOT2 and https://www.ncbi.nlm.nih.gov/gene/?term=GOT1) [27]. The datasets used and/or analyzed during the current study available from the corresponding author on reasonable request.

## Abbreviations

Ang II: Angiotensin II
BSA: Bovine serum albumin
CHD: Congenital heart disease
GOT or AST: Glutamic-oxaloacetic transaminase
HE: Hematoxylin-eosin staining
METTL: Methyltransferase
NADPH: Nicotinamide-adenine dinucleotide phosphate
NCBI: National Center for biotechnology information
PBS: Phosphate-buffered saline
ROS: Reactive oxygen species
SD: Sprague Dawley
TOF: Tetralogy of Fallot
WGA: Wheat germ agglutinin
