# Supplementary material for: Changes in glutamic oxaloacetic transaminase 2 during rat physiological and pathological cardiomyocyte hypertrophy
Source: BMC Cardiovasc Disord. 2023 Dec 5;23:595. doi: 10.1186/s12872-023-03648-3 (PMC10696840; doi:10.1186/s12872-023-03648-3)
Supplement: Supplementary file 1 — Additional file 1. [file 12872_2023_3648_MOESM1_ESM.pptx]

## Slide 1
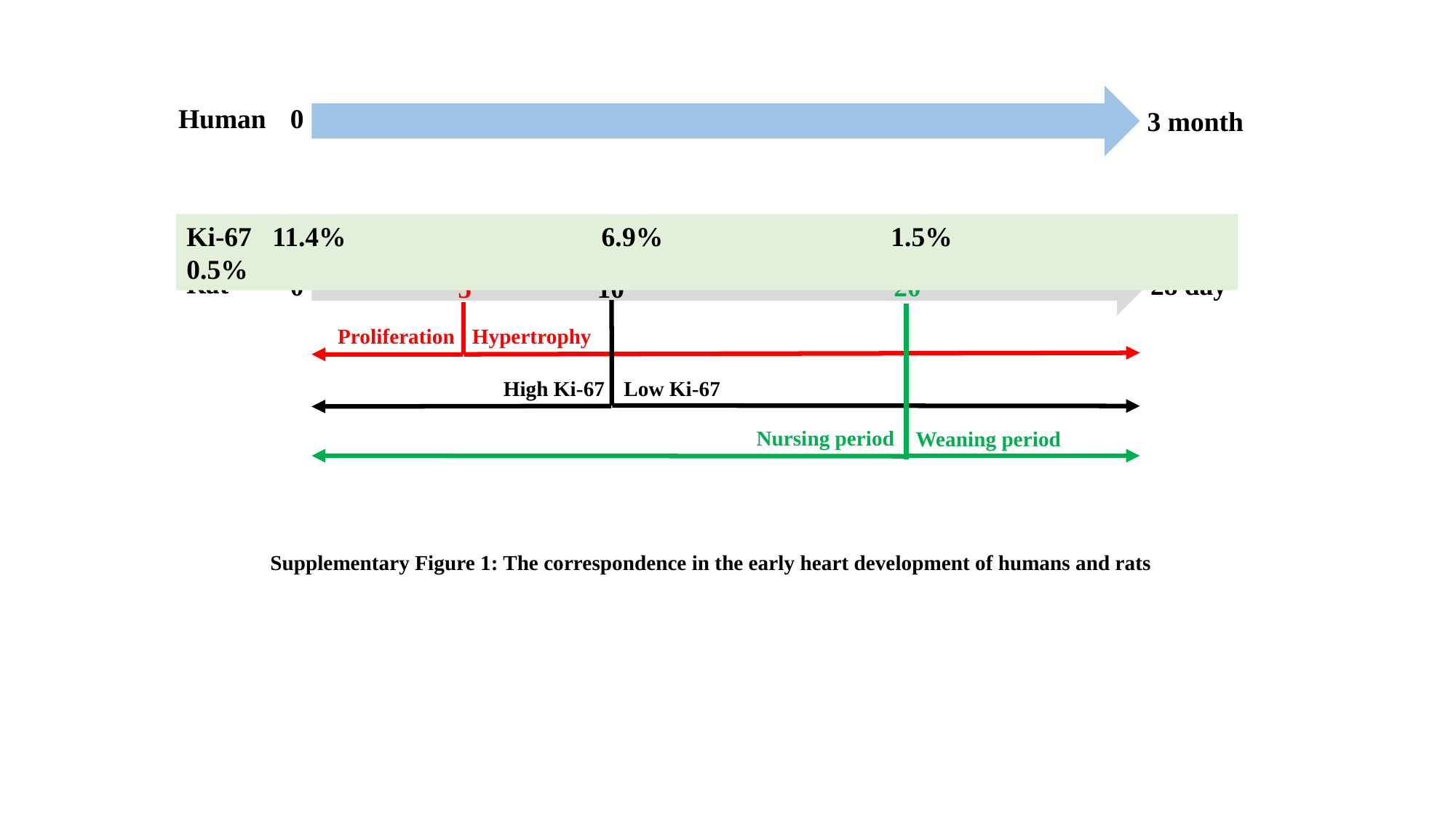

Human
0
3 month
Ki-67 11.4% 6.9% 1.5% 0.5%
Rat
28 day
0
20
5
10
Proliferation
Hypertrophy
Low Ki-67
High Ki-67
Nursing period
Weaning period
Supplementary Figure 1: The correspondence in the early heart development of humans and rats

## Slide 2
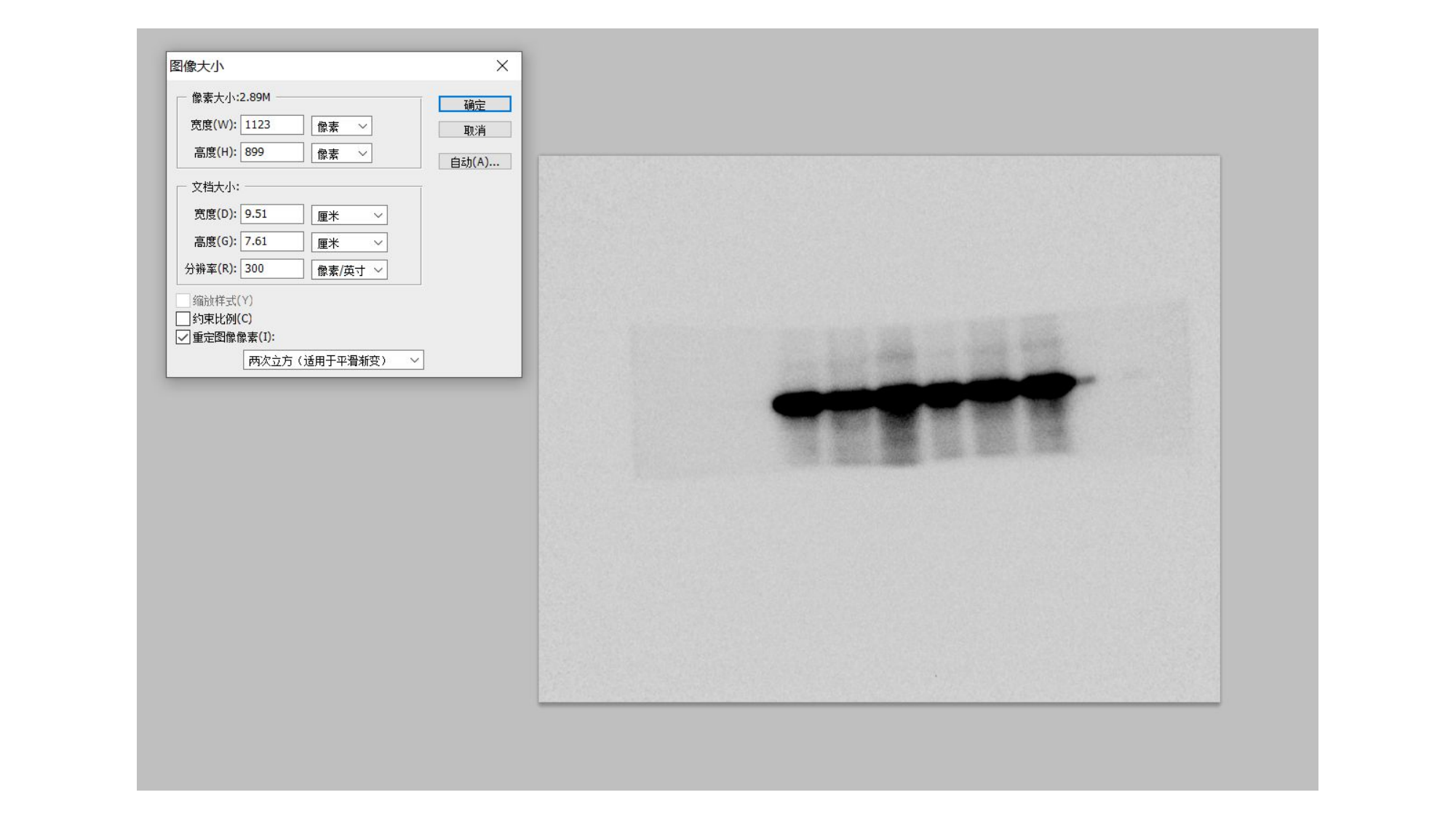

## Slide 3
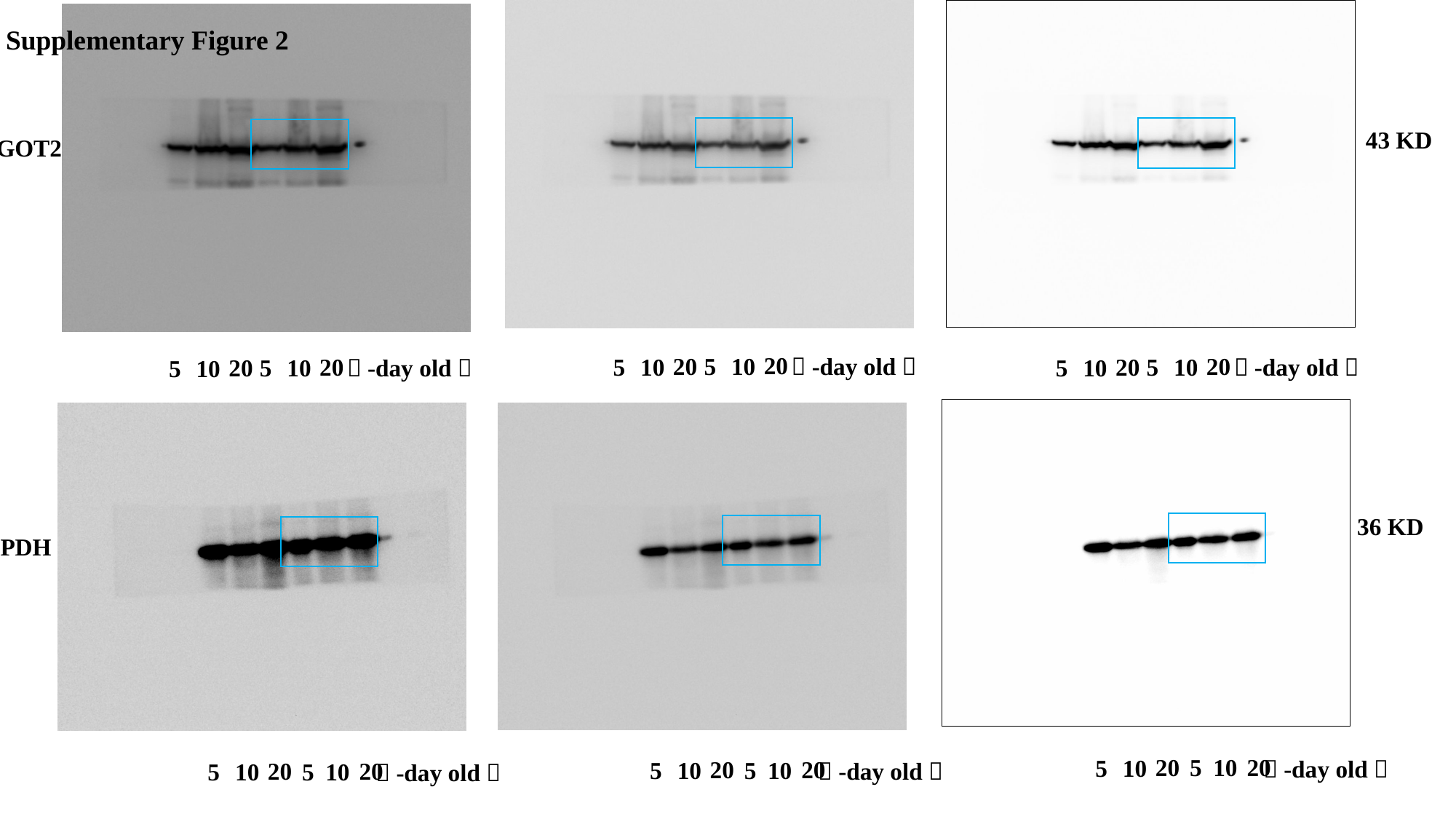

Supplementary Figure 2
43 KD
GOT2
20
5
10
（-day old）
20
20
5
10
（-day old）
5
10
20
20
5
10
5
10
（-day old）
20
5
10
36 KD
GAPDH
20
20
5
10
5
10
（-day old）
20
20
5
10
5
10
20
（-day old）
20
5
10
5
10
（-day old）

## Slide 4
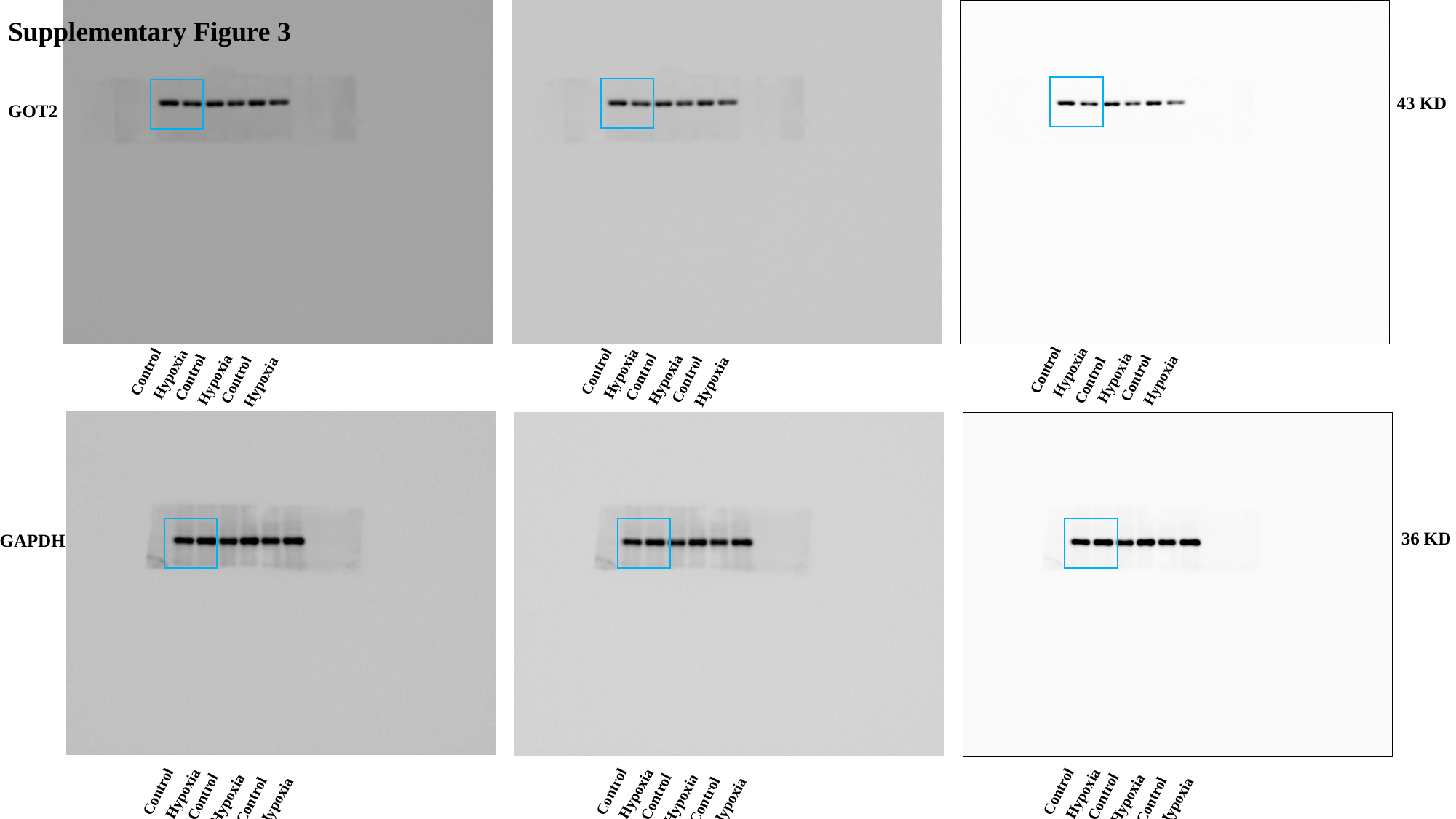

Supplementary Figure 3
43 KD
GOT2
Hypoxia
Hypoxia
Hypoxia
Hypoxia
Control
Hypoxia
Hypoxia
Hypoxia
Control
Control
Hypoxia
Hypoxia
Control
Control
Control
Control
Control
Control
36 KD
GAPDH
Hypoxia
Hypoxia
Hypoxia
Hypoxia
Hypoxia
Hypoxia
Control
Control
Control
Hypoxia
Hypoxia
Hypoxia
Control
Control
Control
Control
Control
Control
